# Characterization of genotype V Japanese encephalitis virus isolates from Republic of Korea

**DOI:** 10.1080/22221751.2024.2362392

**Published:** 2024-05-29

**Authors:** Ah-Ra Lee, Sang-Hyun Kim, Su-Yeon Hong, Sang-Ho Lee, Jae Sang Oh, Kyung Yong Lee, Seong-Jun Kim, Tomohiro Ishikawa, Sang-Mu Shim, Hee Il Lee, Sang-Uk Seo

**Affiliations:** a Department of Biomedicine & Health Sciences, Graduate School, The Catholic University of Korea, Seoul, Republic of Korea; b Department of Microbiology, College of Medicine, The Catholic University of Korea, Seoul, Republic of Korea; c Department of Neurosurgery, Uijeongbu St. Mary's Hospital, College of Medicine, The Catholic University of Korea, Seoul, Republic of Korea; d Division of Cancer Biology, Research Institute, National Cancer Center, Goyang, Republic of Korea; e Center for Infectious Disease Vaccine and Diagnosis Innovation (CEVI), Korea Research Institute of Chemical Technology, Daejeon, Republic of Korea; f Department of Microbiology, Dokkyo Medical University School of Medicine, Tochigi, Japan; g Division of Acute Virus Diseases, Korea National Institute of Health, Korea Disease Control and Prevention Agency, Cheongju, Republic of Korea; h Division of Vectors and Parasitic Diseases, Korea Disease Control and Prevention Agency, Cheongju, Republic of Korea

**Keywords:** Japanese encephalitis virus, genotype V, characterization, virulence, phylogenetic analysis

## Abstract

Japanese encephalitis (JE), caused by the Japanese encephalitis virus (JEV) infection, continues to pose significant public health challenges worldwide despite efficient vaccines. The virus is classified into five genotypes, among which genotype V (GV) was not detected for a long period after its initial isolation in 1952, until reports emerged from China and the Republic of Korea (ROK) since 2009. The characteristics of the virus are crucial in estimating its potential epidemiological impact. However, characterization of GV JEVs has so far been limited to two strains: Muar, the original isolate, and XZ0934, isolated in China. Two additional ROK GV JEV isolates, NCCP 43279 and NCCP 43413, are currently available, but their characteristics have not been explored. Our phylogenetic analysis revealed that GV virus sequences from the ROK segregate into two clades. NCCP 43279 and NCCP 43413 belong to different clades and exhibit distinct *in vitro* phenotypes. NCCP 43279 forms larger plaques but demonstrates inefficient propagation in cell culture compared to NCCP 43413. *In vivo*, NCCP 43279 induces higher morbidity and mortality in mice than NCCP 43413. Notably, NCCP 43279 shows more severe blood–brain barrier damage, suggesting superior brain invasion capabilities. Consistent with its higher virulence, NCCP 43279 displays more pronounced histopathological and immunopathological outcomes. In conclusion, our study confirms that the two ROK isolates are not only classified into different clades but also exhibit distinct *in vitro* and *in vivo* characteristics.

## Introduction

Japanese encephalitis (JE) is a disease caused by infection with the Japanese encephalitis virus (JEV). Although vaccines significantly control the disease [1], JE remains a threat in areas with low vaccine coverage, leading to a substantial number of encephalitis cases [2]. JEV is classified into five genotypes and genotype III (GIII) was prevalent before the 1990s [3]. However, since then, there has been a noticeable surge in genotype I (GI) infections worldwide [4,5]. Therefore, it is plausible that minor genotypes may emerge as the dominant strains in the future. At present, genotype IV (GIV) and genotype V (GV) JEVs causes localized outbreaks. GIV has been specifically identified in Australia since 2022 [6,7], whereas, GV has been detected in China and Republic of Korea (ROK) since 2009 [8–10]. Particularly, data from ROK identified only GV JEVs since 2010, implying that GV may have successfully become the dominant strain in this region [11,12].

Different genotypes of the JEVs can possess distinct molecular and biological characteristics, which might lead to variations in their pathological features. Therefore, characterization of virus is crucial for predicting their potential clinical impact. The Muar and XZ0934, which are the first and second GV JEV isolates have shown higher virulence compared to the currently co-circulating GI and GIII strains [13,14]. This observation suggests that the prevalence of GV could potentially lead to an increased clinical burden.

The necessity for JE vaccination arises from the absence of therapeutic treatments, with current vaccines derived from GIII strains that have offered cross-protective immunity against GI to GIV strains [15–17]. However, immunization with GIII-based vaccines has been shown to yield reduced neutralizing capabilities against Muar, a marked contrast to their effectiveness against GI and GIII strains [18–21]. Further assessments of the efficacy of live or inactivated GIII-based vaccines have revealed only partial protection against XZ0934, significantly less than the full protection observed against GIII strains [20]. These findings highlight a deficiency in current vaccination strategies, emphasizing the urgent need for the development of vaccines specifically targeting GV to bolster defense against this emergent genotype and address the prevention gap in GV JEV infections [22].

While GV JEV has been prevalent for more than a decade, only two isolates are currently available from ROK. K15P38 was derived from a patient's cerebrospinal fluid in 2015 [10], and Sangju was isolated from a collected mosquito pool in 2020 [23]. However, no characterization through cell or animal experiments has been conducted for these isolates. This scarcity of isolates poses a significant challenge in generalizing findings about the GV virus. Therefore, this study aims to observe the characteristics of these two isolates to provide additional information on GV JEV.

## Materials and methods

### Viruses and cells

The two ROK GV JEV strains used in this study were obtained from the National Culture Collection for Pathogens (NCCP). K15P38 and Sangju strains were assigned the codes NCCP 43279 and NCCP 43413, respectively, and will simply be referred to as 43279 and 43413 throughout this paper. The GV JEV Muar strain was generated using the reverse genetic system as previously described [24]. The GIII Nakayama strain was kindly provided by Baik Lin Seong at Yonsei University, ROK. The viral isolates used in this study are listed in Table 1. Baby hamster kidney (BHK)−21 (C-13) cell line was purchased from the Korean Cell Line Bank (KCLB) and maintained in Dulbecco’s Modified Eagle Medium (DMEM) (HyClone, UT, USA) containing 10% fetal bovine serum (FBS) (HyClone, collected and processed in USA) and 1× PS solution (50 U/ml potassium penicillin and 50 µg/ml streptomycin sulfate) (Lonza, MD, USA). All master and working virus stocks used in this study were produced by infecting BHK-21 cells. Viruses used in this study belong to Biosafety Level 2 (BSL-2), and all *in vitro* and *in vivo* experiments involving live viruses were performed in a BSL-2 facility.

**Table 1.** JEV strains used in this study.

Strains	Genotype	Year	Origin	Host	Accession No.	References
Nakayama	GIII	1935	Japan	Homo sapiens	EF571853	Lewis et al. (1947)
Muar	GV	1952	Singapore	Homo sapiens	HM596272	Hale et al. (1952)
43279	GV	2015	ROK	Homo sapiens	PP478074	NCCP
43413	GV	2020	ROK	Mosquito	OR500440	NCCP

### Viral genome sequencing

Viral RNA was directly prepared from virus stock using AccuPrep Viral RNA Extraction Kit (Bioneer, Daejeon, ROK). cDNAs were synthesized using AccuPower RT–PCR PreMix & Master Mix (Bioneer) following the manufacturer’s instruction with some modifications. Sequencing of the amplified cDNA products was performed in both directions, and it was confirmed that the results were consistent when read bidirectionally (Cosmogenetech, Seoul, ROK). The gene sequences of strains 43279 and 43413 have been registered in GenBank with the accession numbers PP478074 and OR500440, respectively.

### Phylogenetic analysis

The reference sequences for both representative GI to GIV JEVs and all GV JEVs, each with a full-length genome, were obtained from the NCBI Reference Sequence Database. All collected data were subjected to statistical analysis for molecular evolution and used to create phylogenetic trees in the Molecular Evolutionary Genetics Analysis 11 (MEGA11) software, utilizing the Maximum Likelihood method and the Tamura-Nei model.

### Virus proliferation and cell cytotoxicity

BHK-21 cells were seeded at a concentration of 4 × 10^5^ cells/well in a 6-well plate. The cells were maintained at 37°C in a 5% CO_2_ incubator for 24 hours prior to infection. Each well was infected with 0.01 MOI of JEV in DMEM containing 2% FBS for 1.5 hours. Subsequently, the wells were washed with Dulbecco’s phosphate-buffered saline (DPBS) (HyClone) and supplemented with 2 ml of DMEM containing 2% FBS. At specific time points, the supernatants were harvested to measure the viral titre. Additionally, adherent cells were counted to evaluate cytotoxicity.

### Mice

5-week-old female BALB/c mice were purchased from Orient Bio (Gyeonggi-do, ROK) and infected intravenously with 10^6^ pfu of JEV. In some experiments, mice were sacrificed 4 days post-infection (DPI) to obtain brain tissue for further analysis. Otherwise, infected mice were monitored for 15 days to assess lethality. The humane endpoint was set at a weight loss of more than 20%. All mice were anesthetized with isoflurane and euthanized by CO_2_ inhalation. All animal research was approved by the Institutional Animal Care and Use Committee at The Catholic University of Korea (approval no. CUMS-2023-0264-01).

### Brain tissue preparation

Euthanized mice were transcardially perfused with 20 ml of cold PBS before their brains were extracted. For the assessment of viral load and inflammatory cytokine production, the brains were mechanically homogenized using the FastPrep-24 5G bead beating system (MP Biomedicals, CA, USA) with a homogenization kit (IGT-25ZS, InnoGeneTech, ROK) in 1 ml PBS. After homogenization, samples were centrifuged at 13,000 rpm for 5 minutes at 4°C, and the supernatants were stored at –80°C until further analysis. In a separate group of mice, to specifically test blood–brain barrier (BBB) permeability and to assess histopathology, the brains were prepared intact, without undergoing any homogenization step, following perfusion.

### Plaque assay

BHK-21 cells were seeded at a concentration of 2.5 × 10^5^ cells/well in a 6-well plate and cultured for 18 hours to assess the viral burden in cell culture supernatant or brain extract. Samples were diluted in DMEM containing 2% FBS and then incubated with cells for 1.5 hours at 37°C in a 5% CO_2_ incubator. The wells were washed with DPBS and received 3 ml of overlay medium containing 2% SeaPlaque agarose (Lonza), 8% FBS, and 1× PS solution in MEM (Welgene, Gyeongsangbuk-do, ROK). After 90 hours of incubation, cells were fixed with 3 ml of 10% formalin (Biosesang, Gyeonggi-do, ROK) for 2 hours. Finally, the wells were stained with a 1% crystal violet solution in 20% ethyl alcohol and thoroughly rinsed with tap water.

### Flow cytometry

The brain was chopped using a clean blade and further digested with RPMI-1640 containing 1 mg/ml of collagenase type 4 (Worthington Biochemical Corporation, NJ, USA) and 50 µg/ml of DNase I (Roche, Basel, Switzerland) for 1 hour at 37°C on shaking incubator. Digested samples were sieved through a 70-µm nylon mesh strainer, and the centrifuged pellets were subjected to Percoll (Cytiva, MA, USA) density gradient centrifugation to isolate mononuclear cells. A total of 1 × 10^5^ mononuclear cells were stained in PBS containing 2% FBS and 2 mM EDTA with combinations of the following antibodies: CD45 (30-F11), CD11b (M1/70), and Ly6G (1A8) purchased from BioLegend (CA, USA); Ly6C (AL-21) purchased from BD Biosciences (CA, USA). After staining, the cells were washed and resuspended in cold PBS containing 2% FBS. Data were analyzed using the BD FACSCanto and FlowJo v10.8.1 software (BD Biosciences).

### Cytokine quantification

Pro-inflammatory cytokine levels in homogenized brain samples were quantified using the Cytometric Bead Array Mouse Inflammation Kit (BD Biosciences) with a BD FACSCanto flow cytometer. Data analysis was subsequently conducted with FCAP Array Software v3.0 (BD Biosciences).

### Histopathology

Brain tissues were fixed in 10% formalin, sectioned at 5 µm, and stained using Hematoxylin and Eosin (H&E). All slides were scanned using the Pannoramic MIDI Scanner (3DHistech, Budapest, Hungary) and analyzed with Case Viewer software (3DHistech). To assess the extent of brain damage, we employed a scoring system ranging from 0 to 4, with each level corresponding to the severity of observed histological changes. Score 0 (Normal) brain tissue shows a normal appearance with intact outer molecular layer, external granular layer, and external pyramidal layer. Score 1 (Mild) is characterized by abnormal destruction in cortical layers, invasion of granular cells into the outer molecular cortex layers, and shrunken cells with pyknotic nuclei. Score 2 (Moderate) includes observations noted in the Mild category, plus the presence of extravasated blood and clearly dilated, congested blood vessels. Score 3 (Severe) involves extensive destruction and abnormal irregularities in the outer cortical layers, with cell infiltration, perivascular hemorrhage, and necrotic lesions surrounding shrunken cells with pyknotic nuclei. Score 4 (Terminal) encompasses all pathological features observed in the other categories, alongside extremely critical damage across a larger area of the tissue.

### BBB permeability assay

Mice were intravenously injected with 3 ml/kg of 2% Evans blue (EB) at 4 DPI. Three hours after EB injection, the brains were extracted and analyzed for BBB penetration using digital imaging analysis with ImageJ software. The EB-positive areas were quantified with specific colour thresholding conditions (Hue: 0-107, Saturation: 50-255, Brightness: 0-226) and utilizing the black/white inversion feature.

### Statistical analysis

In all experiments, individual values are presented as the mean and standard deviation (SD). All data were processed using GraphPad Prism v10.1.2 (GraphPad, CA, USA). Statistical analyses were performed using Student's t-test (two-tailed), analysis of variance (ANOVA) with Tukey's multiple comparisons test, and the Kaplan-Meier methods. Statistical significance was determined at a *p*-value of less than 0.05, denoted as follows: *p < 0.05; **p < 0.01; ***p < 0.001; ****p < 0.0001.

## Results

### Sequence analysis of GV isolates from ROK

**Figure 1.**
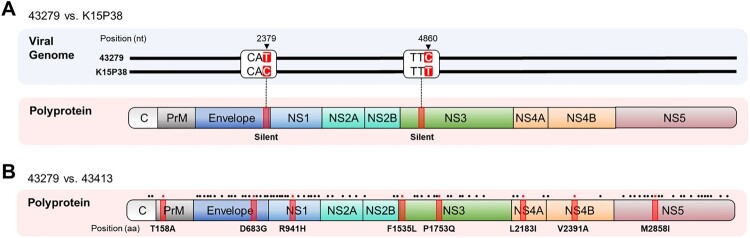
**Nucleotide and amino acid sequence comparison of 43279 with K15P38 and 43413. (A)** Viral genome sequences (upper panel) and amino acid sequences of polypeptide (lower panel) were compared between strains 43279 and K15P38. Mutations involving silent mutations and the corresponding amino acid positions are highlighted in red. Position numbers are calculated using only the open reading frame (ORF), omitting the untranslated regions. **(B)** Amino acid sequences of polypeptides were compared between strains 43279 and 43413. The amino acid positions related to gene mutations are indicated by dots at the top of the diagram, and positions with amino acid substitutions are highlighted in red.

We sequenced the entire open reading frames (ORFs) of two deposited GV JEV isolates, 43279 and 43413, and compared their sequences with those of the previously characterized isolates, Muar and XZ0934 (Table 2 and Supplementary Table 1). We also included the full sequence of K15P38 previously reported in an original clinical study, which corresponds to the deposited designation of our analyzed strain 43279. This comparison revealed that 43279 exhibited a single nucleotide difference in both the *Env* and *Ns3* genes relative to K15P38, yet the amino acid sequences were 100% identical (Figure 1(A)). The genetic sequence of 43279 exhibited homologies of 90.4% and 97.4% with Muar and XZ0934, respectively, whereas the amino acid sequences showed similarities of 98.3% and 99.5%, respectively (Table 2). The comparison between strains 43279 and 43413 showed nucleotide and amino acid sequence homologies of 99.2% and 99.8%, respectively, Although there were 86 nucleotide differences, only 8 resulted in amino acid substitutions (Figure 1(B) and Supplementary Table 1). These results highlight the higher degree of similarity among ROK isolates compared to isolates from other countries.

**Table 2.** Nucleotide and amino acid sequence identities of GV JEV strains.

Strains		Sequence identity (%) of nucleotides (amino acids) in gene segments										
		ORF	C	prM	E	NS1	NS2A	NS2B	NS3	NS4A	NS4B	NS5
43279 vs.	Muar	90.4 (98.3)	92.7 (93.7)	93.6 (99.4)	90.0 (98.8)	89.5 (98.9)	91.0 (99.1)	89.8 (99.2)	90.6 (98.2)	89.5 (99.3)	88.4 (95.7)	90.5 (98.5)
	XZ0934	97.4 (99.5)	98.4 (98.4)	98.0 (100)	97.1 (99.6)	97.1 (99.4)	97.8 (100)	97.7 (99.2)	97.0 (99.4)	96.9 (98.7)	98.0 (99.2)	97.6 (99.8)
	K15P38	100 (100)	100 (100)	100 (100)	99.9 (100)	100 (100)	100 (100)	100 (100)	99.9 (100)	100 (100)	100 (100)	100 (100)
	43413	99.2 (99.8)	99.5 (100)	99.8 (99.4)	98.8 (99.8)	98.7 (99.7)	99.3 (100)	99.2 (100)	99.2 (99.7)	99.1 (99.3)	99.6 (99.6)	99.2 (99.9)
43413 vs.	Muar	90.4 (98.3)	92.1 (93.7)	93.4 (98.8)	89.9 (98.6)	90.0 (98.6)	91.2 (99.1)	90.1 (99.2)	90.6 (98.5)	89.5 (100)	88.5 (96.1)	90.5 (98.3)
	XZ0934	97.5 (99.6)	98.4 (98.4)	97.8 (99.4)	97.1 (99.4)	97.3 (99.1)	97.8 (100)	97.5 (100)	97.4 (99.7)	96.9 (99.3)	97.9 (99.6)	97.7 (99.9)
	K15P38	99.2 (99.8)	99.5 (100)	99.8 (99.4)	98.9 (99.8)	98.7 (99.7)	99.3 (100)	97.5 (99.2)	99.2 (99.7)	99.1 (99.3)	99.6 (99.6)	99.2 (99.9)
	43279	99.2 (99.8)	99.5 (100)	99.8 (99.4)	98.8 (99.8)	98.7 (99.7)	99.3 (100)	99.2 (100)	99.2 (99.7)	99.1 (99.3)	99.6 (99.6)	99.2 (99.9)

### Phylogenetic analysis of GV isolates

**Figure 2.**
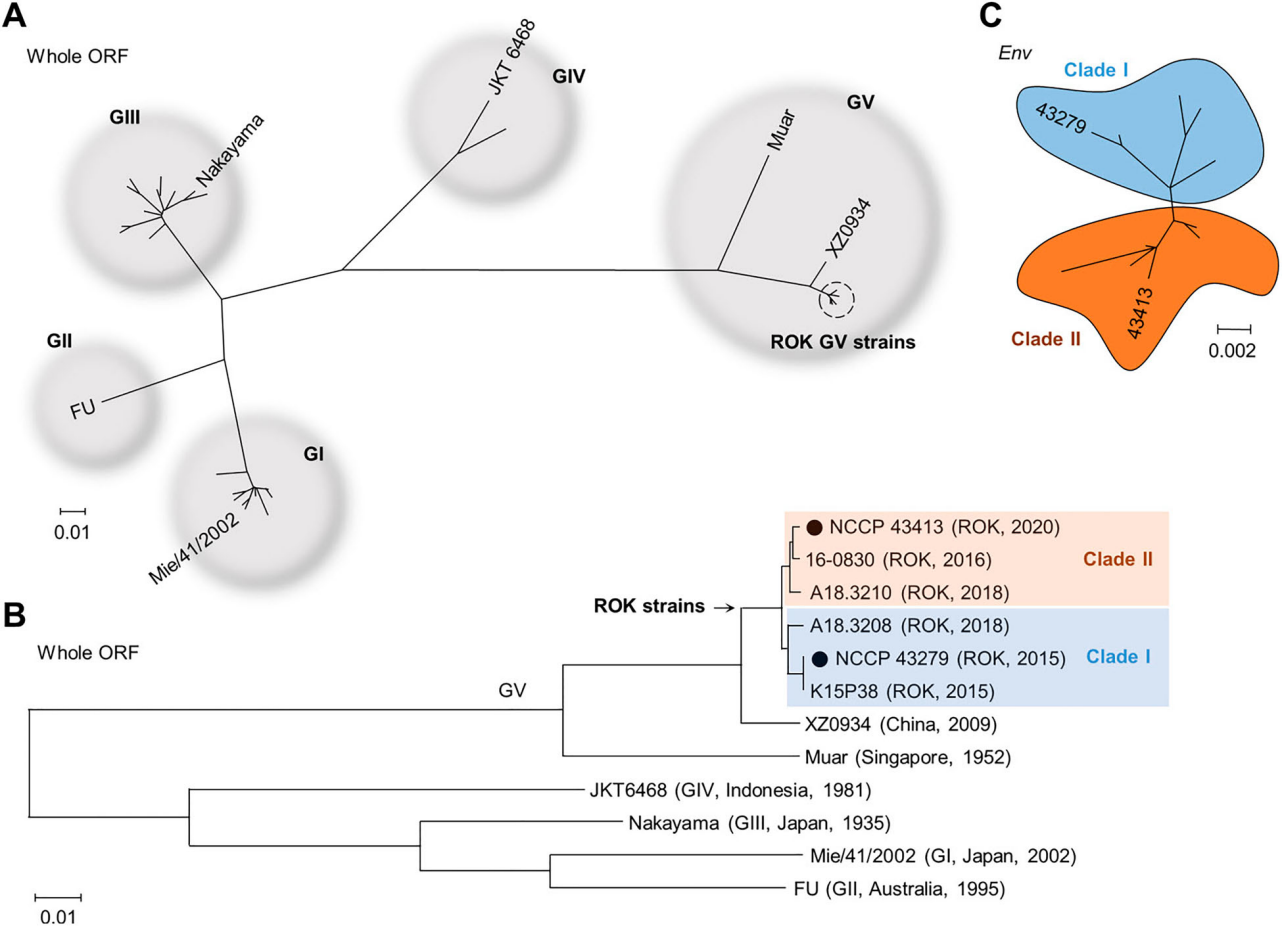
**Phylogenetic analysis of 43279 and 43413.** Phylogenetic analysis was conducted using maximum likelihood (Tamura-Nei model) analysis. **(A)** The analysis utilized the whole open reading frame (ORF) sequences of all JEV genotypes, incorporating a total of 31 whole ORF sequences. **(B)** A phylogenetic analysis on the whole ORF sequences of GV JEVs and representative strains from genotypes GI to GIV, with 43279 and 43413 marked with closed circles. **(C)** A cluster analysis of GV JEVs from the ROK was performed based on 15 *Env* gene sequences. Sequences from distinct clades are highlighted in different colours. The scale bar represents estimated evolutionary distances with 0.01 (A, B) or 0.002 (C) changes per nucleotide position.

We conducted a phylogenetic analysis including all reported GV JEV whole ORF sequences, along with representative sequences from GI to GIV. As expected, the sequences of GV JEV reported from the ROK grouped separately from Muar and XZ0934 (Figure 2(A)). Importantly, the ROK isolates could be further divided into two distinct clades, with 43279 and 43413 each belonging to different clades (Figure 2(B)). Similarly, analysis of the *Env* gene, which has been more frequently reported for GV JEVs, confirmed that the ROK isolates can be classified into two distinct clades (Figure 2(C)).

Amino acid sequence changes are directly associated with the virus characteristics. Therefore, we confirmed the amino acid changes present in GV isolates from the ROK (Table 3). Previous studies targeting the Envelope (E) amino acid sequence reported an R84K substitution unique to ROK isolates [10]. In our analysis, we identified four additional ROK-specific substitutions: non-structural (NS)1 (105A), NS3 (210K, 249P, 269K). Notably, while NS3 249P was observed in most viruses reported in ROK, isolate 43413 possessed 249Q, identical to Muar and XZ0934. Furthermore, amino acid sequence differences specific to the clades of ROK strains were identified in NS1 at position 147, NS3 at 31, NS4A at 60, and NS5 at 330.

**Table 3.** Amino acid substitutions in GV JEV strains.

			Position											
Strains	Host	Year	prM	E	E	NS1	NS1	NS3	NS3	NS3	NS3	NS4A	NS4B	NS5
			31	84^a^	389	105^a^	147^b^	31^b^	210^a^	249^a^	269^a^	60^b^	119	330^b^
Muar	Human	1952	T	R	D	S	R	L	R	Q	R	I	A	M
K15P38^c^	Human	2015	T	K	D	A	R	F	K	P	K	L	V	M
43279^c^	Human	2015	T	K	D	A	R	F	K	P	K	L	V	M
A18.3208^c^	Mosquito	2018	T	K	D	A	R	F	K	P	K	L	A	M
16-0830^c^	Mosquito	2016	T	K	D	A	H	L	K	P	K	I	A	I
A18.3210^c^	Mosquito	2018	T	K	D	A	H	L	K	P	K	I	A	I
43413^c^	Mosquito	2020	A	K	G	A	H	L	K	Q	K	I	A	I
XZ0934	Mosquito	2009	T	R	D	T	R	L	R	Q	R	I	A	I

^a^ ROK isolate-specific substitutions; ^b^Clade-specific substitutions; ^c^ROK isolates.

### In vitro characterization of 43279 and 43413

**Figure 3.**
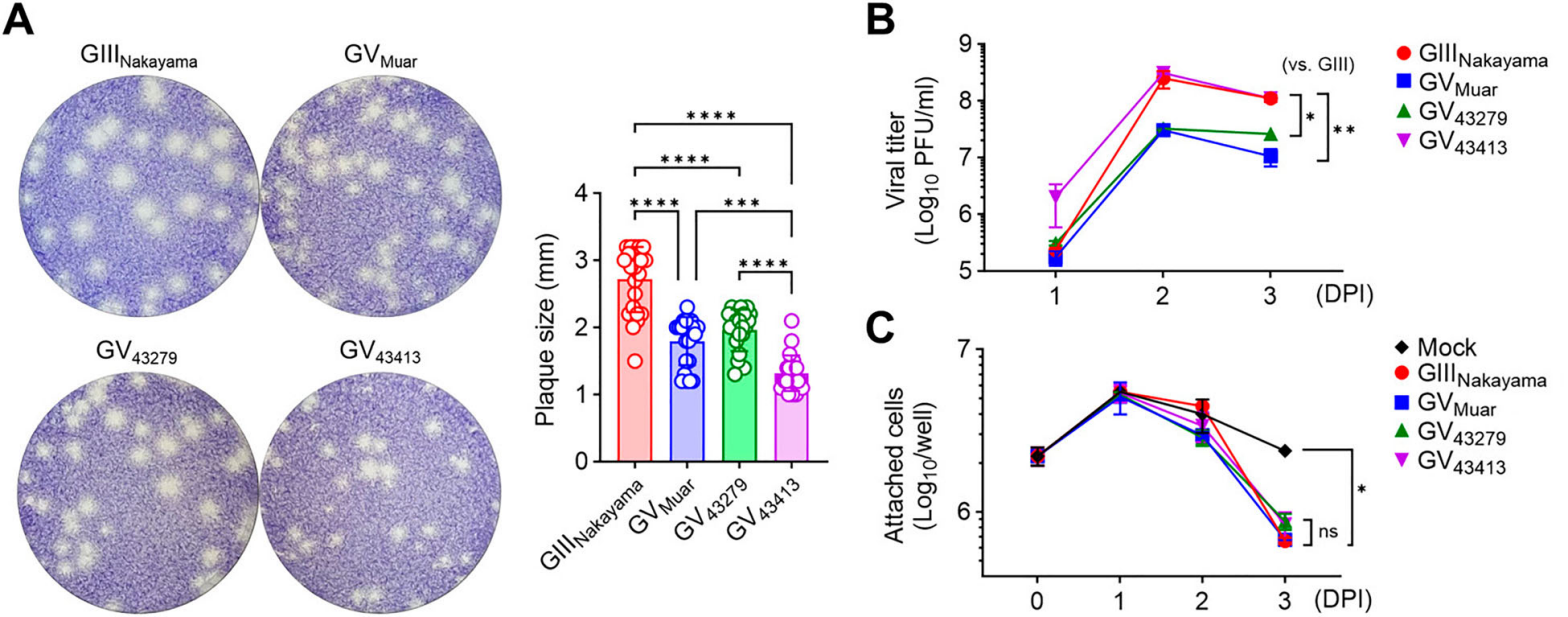
***In vitro* characterization of 43279 and 43413. (A)** BHK-21 cells were cultured in a 6-well plate and infected with the indicated JEVs. Plaque sizes were measured at 70 hours post-infection (n = 23). Every dot represents the mean plaque size in each well. **(B, C)** BHK-21 cells were infected with JEVs at an MOI of 0.01 (n = 6). Virus titre (B) and the number of attached cells (C) were measured at indicated time points. Data were pooled from three independent experiments.

To investigate the effects of previously discussed phylogenetic distinctiveness and amino acid sequence changes on the characteristics of ROK isolates *in vitro*, we examined virus propagation and cytotoxicity in cell lines infected at the same MOI. We utilized the BHK-21 cell line for *in vitro* experiments as this cell line facilitates virus propagation and is advantageous for observing cytopathic effects and plaque formation [25]. Our results indicated that 43279 formed plaques similar in size to those of Muar and smaller than those formed by the GIII Nakayama strain (Figure 3(A)). 43413 formed even smaller plaques compared to both Muar and 43279 (Figure 3(A)). When virus titres in the culture supernatant were measured at 1, 2, and 3 DPI, Muar and 43279 exhibited lower titres compared to Nakayama (Figure 3(B)). However, 43413 showed a propagation trend similar to Nakayama (Figure 3(B)). While plaque size and titres differed between 43279 and 43413, the cytotoxicity, as measured by the number of adherent cells, was comparable across all viruses tested in the experiment (Figure 3(C)).

### In vivo characterization of 43279 and 43413

**Figure 4.**
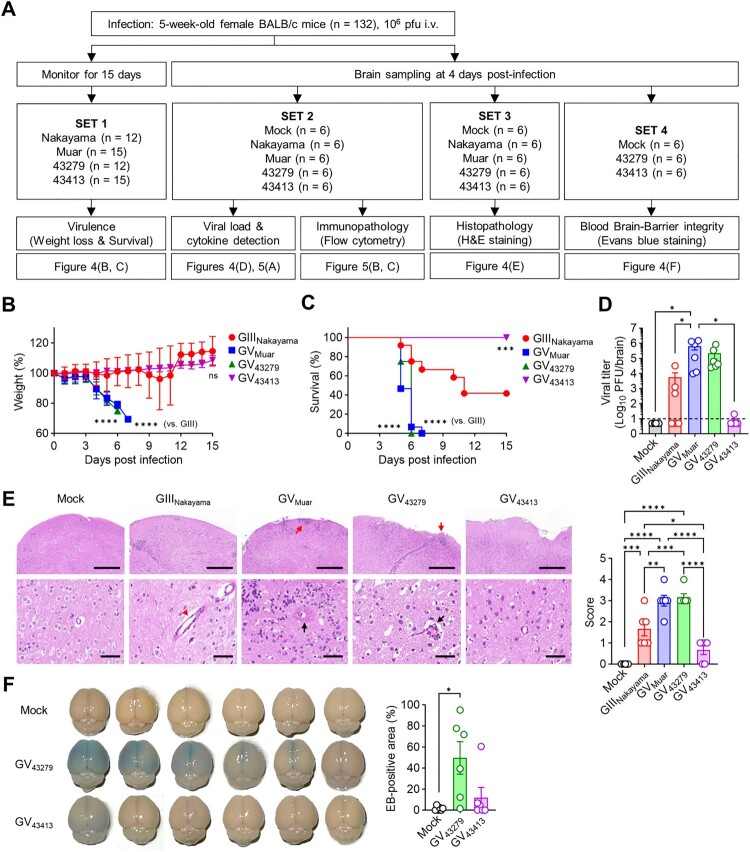
***In vivo* characterization of 43279 and 43413 in mice**. **(A)** Flowchart illustrating the animal experiments conducted in this study. BALB/c mice were intravenously infected with 10^6^ pfu of each JEV strain. **(B, C)** Weigh changes (B) and survival rates (C) were monitored for 15 days (n = 12-15). Data were pooled from three independent experiments. **(D-F)** Sets of BALB/c mice were infected with 10^6^ pfu of each JEV strain and sacrificed for virus titration (D), evaluation of histological pathology (E), and BBB integrity test (F) in brain tissue (n = 6, each set). Data were pooled from two independent experiments. **(E)** Invasion of abnormal granular cells into outer molecular cortex layers (red arrow), extravasation of blood (red arrow head), and perivascular hemorrhage with massive cell infiltration (black arrow) are indicated. Scale bars = 500 µm (upper panel), 50 µm (lower panel).

Next, we conducted a series of mouse experiments for the characterization of ROK isolates *in vivo* (Figure 4(A)). To investigate virulence, mice were infected with the same titre of virus and observed the progression of disease over 15 days (Figure 4(B, C)). Previous studies have indicated that GV JEVs exhibit higher virulence than GIII in mice [21,26]. In our experiments, Mice infected with Muar showed a significant weight loss and higher mortality compared to the GIII Nakayama. Similarly, mice infected with 43279 displayed weight loss and mortality rates comparable to Muar. However, mice infected with 43413 showed a gradual increase in weight, and all mice eventually survived. This trend was also reflected in the viral burden in the brain; mice infected with Muar and 43279 exhibited higher viral titres compared to those infected with Nakayama. Conversely, mice infected with 43413 showed very low levels of virus detection (Figure 4(D)).

Examination of H&E stained brain tissue (Figure 4(E)) also revealed that infections with Muar (score 3.00 ± 0.53) and 43279 (score 3.17 ± 0.35) resulted in severe destruction and abnormal irregularity of the outer cortical layer with cell infiltration. These samples also showed perivascular hemorrhage and necrotic lesions around shrunken cells with pyknotic nuclei in the internal granular and pyramidal layers. Notably, perivascular cuffing and a massive presence of perivascular cells were observed in the destroyed cortex layer. This pathology is characterized by most cells exhibiting deeply stained nuclei with a surrounding clear halo, which is indicative of significant brain edema. Such findings highlight the extent of cerebral damage and swelling, potentially leading to fatal outcomes. Infections with Nakayama (score 1.67 ± 0.69) exhibited a more moderate histopathology but clearly showed extravasation of blood and dilated congested blood vessels. As anticipated, strain 43413 (score 0.67 ± 0.44) demonstrated only mild brain damage.

JEV can infect the brain more efficiently through compromising BBB integrity [27,28]. To test whether the distinct virulence between strains 43279 and 43413 was due to differences in the degree of BBB permeability contributing to brain infection, we injected EB into the bloodstream of infected mice at 4 DPI and detected it in the brain (Figure 4(F)). The results showed that mice infected with 43279 had a more noticeable EB influx in the brain. However, consistent with previous *in vivo* data, only one out of six mice infected with 43413 displayed obvious EB detection.

### Immunopathological characterization of 43279 and 43413

**Figure 5.**
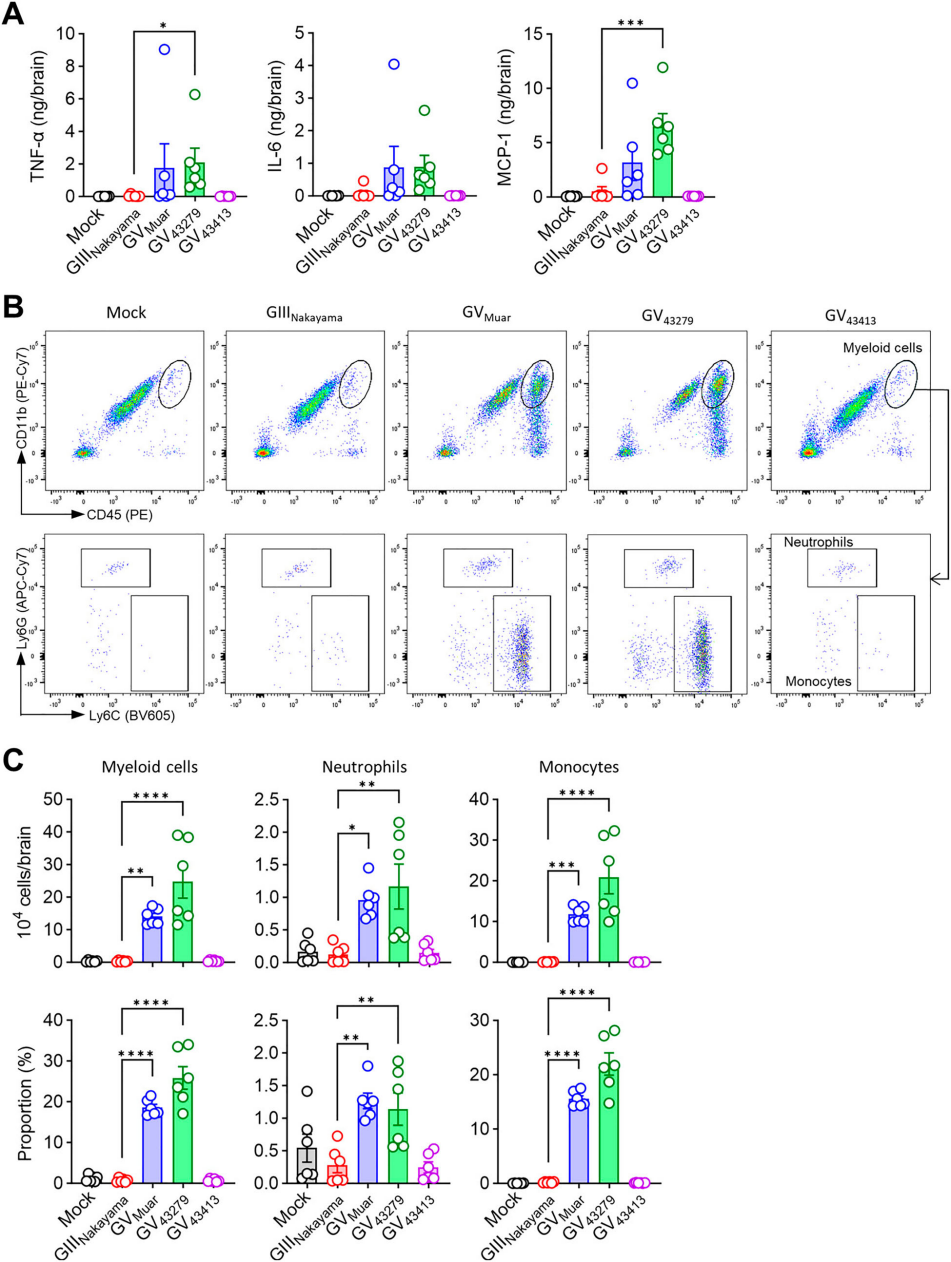
**Immunopathology of mice infected with 43279 and 43413.** BALB/c mice were intravenously infected with 10^6^ pfu of each JEV strain (n = 6). **(A)** TNF-α, IL-6, and MCP-1 levels were measured in brain homogenates (n = 6). **(B)** Representative flow cytometry analysis of brain myeloid cells (CD45^hi^CD11b^+^). Myeloid cells were further divided into neutrophils (Ly6C^int^Ly6G^+^) and monocytes (Ly6C^hi^Ly6G^-^). **(C)** Absolute number and proportion of myeloid cells in the brain (n = 6). Data were pooled from two independent experiments.

We subsequently examined the immunopathology in brains of mice infected with JEVs. Brains infected with the high-virulence strains, Muar and 43279, exhibited an increased presence of inflammation-related cytokines, including TNF-α, IL-6, and MCP-1 (Figure 5(A)). Neutrophils (CD11b^+^Ly6C^int^Ly6G^+^) and monocytes (CD11b^+^Ly6C^hi^Ly6G^-^) are key innate immune cells that infiltrate infected organs, serving as hallmarks of infection [29,30]. Using flow cytometry, we confirmed the influx of these two cell types (Figure 5(B)). Our analysis revealed a statistically significant increase in the influx of myeloid cells (CD45^hi^CD11b^+^) in the brains of mice infected with Muar and 43279, compared to those infected with Nakayama and 43413 (Figure 5(C)). This elevation in myeloid cells was attributed to increases in both neutrophils and monocytes, with a notably higher influx of monocytes.

## Discussion

In this study, we used the NCCP codes, 43279 and 43413, as strain names. GV JEV isolates from ROK are exclusively available through NCCP, and our sequencing revealed that these viruses possess distinct mutations compared to the K15P38 or Sangju strains, indicating the necessity to consider them as separate entities. Despite 43279 showed no substitutions in the amino acid sequence, it differed by one nucleotide in both the *Env* and *Ns3* gene sequences compared to K15P38. Although these differences seem minor, variations in its genome sequence could have influenced the RNA secondary structure or codon usage, potentially affecting the replication cycle [31–33]. Sangju has been fully sequenced only for the *Env* gene, and when compared to 43413, the differences are more significant than those between 43279 and K15P38. There were a total of 17 nucleotide differences between the *Env* gene sequence of 43413 and Sangju, demonstrating 98.9% homology but resulting in only one D389G amino acid substitution. Given these differences, it is difficult to consider 43413 as identical to Sangju for the purpose of this research.

Our analysis suggests that the viruses circulating in the ROK are divided into two clades, and we identified four amino acid substitutions to be clade-specific. Each clade has unique amino acid sequences at these four positions, and these sequences match either Muar or XZ0934. Intriguingly, at the 330 position in NS5, Clade I possesses 330M, identical to Muar, while Clade II has 330I, identical to XZ0934. The fact that viruses from different clades share different sequences from Muar and XZ0934 is difficult to attribute to coincidence. There are two possible explanations: first, the M330I substitution might be a beneficial mutation, leading descendants of Muar to be introduced into the ROK and undergo the same M330I substitution as XZ0934; second, descendants of Muar and XZ0934 might have independently introduced into the ROK, forming separate clades. While the exact mechanisms remain elusive with the data presently available, additional investigations into this substitution are imperative to unravel the underlying reasons for the bifurcation of ROK viruses into two distinct clades. A study published by Sanborn et al. in 2021 reported the detection of GV JEV sequences through metagenomic analysis of mosquito pools in the ROK [34]. This research, which included the full genome sequencing of A18.3208 (clade I) and A18.3210 (clade II) isolated from different mosquito pools, demonstrated that viruses belonging to different clades were co-circulating in the ROK.

Previous studies comparing GV isolates such as Muar and XZ0934 with other genotypes of JEV have shown variable results depending on experimental conditions. Particularly *in vitro*, differences were observed based on the type of cell line or the virus strain used for comparison. For example, GV strains typically showed similar propagation kinetics to GI and GIII JEVs in most cell lines but exhibited inefficient growth in neuroblastoma cells [14,26]. However, our results indicated that Muar exhibited lower *in vitro* propagation compared to Nakayama, even in BHK-21 fibroblast cells. Similar inconsistencies have also been observed in plaque formation experiments. XZ0934 formed smaller plaques than GIII strains in two independent studies [20,21], while Muar produced plaques of similar size to GIII Beijing [14]. Nonetheless, Muar still formed smaller plaques than GI Mie/41/2002 or GIV 19CxBa-83-Cv [14,26]. In our study, all tested GV isolates showed smaller plaques compared to Nakayama. As demonstrated, the variability of *in vitro* experimental results, influenced by specific conditions, points out the challenges in fully elucidating virus pathology through studies based on cell lines. Nonetheless, such data underscore the potential functional significance of genomic and amino acid sequence disparities observed among GV isolates. Given the critical role of cell lines in virus propagation and titration, these characterizations remain fundamentally important. *In vivo* experiments with mice also showed variability depending on the mouse or virus strain used, but generally, GV JEV strains were found to be more virulent than GI, GIII, and GIV strains [14,20,21,26]. Consistently, we observed that Muar and the ROK isolate 43279 exhibited high virulence compared to GIII JEV, Nakayama. However, as our study has confirmed that 43279 and 43413, each representing a distinct clade, exhibit markedly different virulence, further investigation is required to determine if other co-circulating viruses also exhibit clade-specific differences in both *in vivo* and *in vitro* settings.

According to our analysis, strains 43279 and 43413 differ by a total of eight amino acid sequences. The mismatches in amino acid sequences between the two strains are distributed across various proteins, except for two instances in NS3, complicating predictions about which substitution is most critical to the observed phenotypic differences. Although we have not defined the substitution that plays a decisive role in the attenuated phenotype of 43413, we observed that 43413 was not able to effectively reduce BBB permeability at early stages (i.e. 4 DPI), suggesting a mechanism that reduces the virulence of 43413. Delayed BBB disruption by 43413 may allow for the efficient engagement of adaptive immunity, potentially resulting in a lower viral burden, and reduced brain histopathology. Recent studies have reported that the NS1 protein of JEV disrupts the BBB [28]. While further research is necessary, considering our amino acid sequence data, the observed R941H substitution in NS1 could potentially contribute to the attenuated virulence of strain 43413.

Differences in amino acid sequences in structural proteins such as premembrane (prM) and E affect their antigenic characteristics. Our previous studies have shown that amino acid substitution at position 389 of the E protein significantly affects the neutralizing antibody response [22]. Additionally, 43413 possesses 31A in the prM protein, unlike other GV viruses which have 31T. This difference may also contribute to the antigenic differences between these viruses. The identification of such variations is essential for vaccine development, as the detailed sequence analysis of structural proteins of circulating viruses is necessary for selecting the most effective antigenic sequences. Moreover, the presence of 84K in the E protein among ROK isolates, which is absent in GV isolates from other regions, is significant and could potentially impact vaccine efficacy.

Contrasting with the attenuated phenotype of strain 43413, mice infected with high virulence strains, such as 43279 and Muar, exhibited markedly different immune responses during the early phases of infection. This includes both influx of myeloid cells and increased neurotoxic inflammatory cytokines. While there was no significant change in the number of microglia, the most pronounced increase was observed in monocyte and neutrophil which originated from hematopoietic organs like the bone marrow. The accumulation of monocytes and neutrophils is associated with various types of brain pathology [29,30]. Furthermore, these cells are the primary groups responsible for producing inflammatory mediators. Eventually, the infiltration of myeloid cells serves to amplify inflammation through the sustained activity of innate immune cells. Notably, our analysis indicated that the influx of monocytes was more pronounced than that of neutrophils. Furthermore, the recruitment of monocytes is facilitated by chemokines such as MCP-1, and our results also suggest that an increase in MCP-1 levels coincides with the increased influx of monocytes in cases infected with high virulence strains. Some studies have reported that treatment with CCR2 inhibitors, among other interventions, can alleviate pathology in brain diseases [35]. It will be important to further investigate whether the influx of cells such as monocytes can regulate overall pathology in JEV infections through additional research, potentially leading to novel antiviral strategies.

Through this research, we explored the phylogenetic relationships and potential pathological characteristics of the 43279 and 43413 strains. Although our findings offer insights that could support efforts against genotype displacement by GV in JEV endemic areas, the investigation was still limited to a small selection of isolates. Hence, securing and analyzing additional isolates is imperative. Moreover, employing reverse genetics technology to identify key substitutions linked to observed phenotypes is crucial for a deeper understanding of JEV virulence.

## Supplementary Material

Supplemental Material
