# Supplementary material for: Characterization of genotype V Japanese encephalitis virus isolates from Republic of Korea
Source: Emerg Microbes Infect. 2024 May 29;13(1):2362392. doi: 10.1080/22221751.2024.2362392 (PMC11168223; doi:10.1080/22221751.2024.2362392)
Supplement: Supplemental Material [file TEMI_A_2362392_SM3500.docx]

**Supplementary Material for**

**Characterization of Genotype V Japanese Encephalitis Virus Isolates from Republic of Korea**

Ah-Ra Lee^a,b^, Sang-Hyun Kim^a,b^, Su-Yeon Hong^a,b^, Sang-Ho Lee^a,b^, Jae Sang Oh^c^,

Kyung Yong Lee^d^, Seong-Jun Kim^e^, Tomohiro Ishikawa^f^, Sang-Mu Shim^g^,

Hee Il Lee^h^, and Sang-Uk Seo^a,b^

^a^Department of Biomedicine & Health Sciences, Graduate School, The Catholic University of Korea, Seoul, Republic of Korea, ^b^Department of Microbiology, College of Medicine, The Catholic University of Korea, Seoul, Republic of Korea, ^c^Department of Neurosurgery, Uijeongbu St. Mary's Hospital, College of Medicine, The Catholic University of Korea, Seoul, Republic of Korea, ^d^Division of Cancer Biology, Research Institute, National Cancer Center, Goyang, Republic of Korea, ^e^Center for Infectious Disease Vaccine and Diagnosis Innovation (CEVI), Korea Research Institute of Chemical Technology, Daejeon, Republic of Korea, ^f^Department of Microbiology, Dokkyo Medical University School of Medicine, Tochigi, Japan, ^g^Division of Acute Virus Diseases, Korea National Institute of Health, Korea Disease Control and Prevention Agency, Cheongju, Republic of Korea, ^h^Division of Vectors and Parasitic Diseases, Korea Disease Control and Prevention Agency, Cheongju, Republic of Korea.

Corresponding author: Sang-Uk Seo, suseo@catholic.ac.kr, The Catholic University of Korea, Seoul, Republic of Korea

**Supplementary Table 1.** Number of nucleotide and amino acid sequence variations in GV JEV strains.

| Strains | | Number of sequence variants of nucleotides (amino acids) in gene segments | | | | | | | | | | | |
| --- | --- | --- | --- | --- | --- | --- | --- | --- | --- | --- | --- | --- | --- |
|  |  | ORF | C | PrM | E | NS1 | NS2A | NS2B | NS3 | NS4A | NS4B | NS5 |  |
| 43279  vs. | Muar | 990  (59) | 28  (8) | 32  (1) | 150  (6) | 111  (4) | 61  (2) | 40  (1) | 175  (11) | 47  (1) | 89  (11) | 257  (14) |  |
|  | XZ0934 | 264  (17) | 6  (2) | 10  (0) | 43  (2) | 31  (2) | 15  (0) | 9  (1) | 55  (4) | 14  (2) | 15  (2) | 66  (2) |  |
|  | K15P38 | 2  (0) | 0  (0) | 0  (0) | 1  (0) | 0  (0) | 0  (0) | 0  (0) | 1  (0) | 0  (0) | 0  (0) | 0  (0) |  |
|  | 43413 | 86  (8) | 2  (0) | 1  (1) | 18  (1) | 14  (1) | 5  (0) | 3  (0) | 15  (2) | 4  (1) | 3  (1) | 21  (1) |  |
| 43413  vs. | Muar | 987  (59) | 30  (8) | 33  (2) | 152  (7) | 106  (5) | 60  (2) | 39  (1) | 174  (9) | 47  (0) | 88  (10) | 258  (15) |  |
|  | XZ0934 | 255  (15) | 6  (2) | 11  (1) | 43  (3) | 29  (3) | 15  (0) | 10  (1) | 48  (2) | 14  (1) | 16  (1) | 63  (1) |  |
|  | K15P38 | 84  (8) | 2  (0) | 1  (1) | 17  (1) | 14  (1) | 5  (0) | 3  (0) | 14  (2) | 4  (1) | 3  (1) | 21  (1) |  |
|  | 43279 | 86  (8) | 2  (0) | 1  (1) | 18  (1) | 14  (1) | 5  (0) | 3  (0) | 15  (2) | 4  (1) | 3  (1) | 21  (1) |  |
